# Functional Characterization of Preadipocytes Derived from Human Periaortic Adipose Tissue

**DOI:** 10.1155/2017/2945012

**Published:** 2017-10-25

**Authors:** Diana Vargas, Jaime Camacho, Juan Duque, Marisol Carreño, Edward Acero, Máximo Pérez, Sergio Ramirez, Juan Umaña, Carlos Obando, Albert Guerrero, Néstor Sandoval, Gina Rodríguez, Fernando Lizcano

**Affiliations:** ^1^Center of Biomedical Investigation Universidad de La Sabana (CIBUS), Chía, Colombia; ^2^Fundación Cardioinfantil-Instituto de Cardiología, Bogota, Colombia

## Abstract

Adipose tissue can affect the metabolic control of the cardiovascular system, and its anatomic location can affect the vascular function differently. In this study, biochemical and phenotypical characteristics of adipose tissue from periaortic fat were evaluated. Periaortic and subcutaneous adipose tissues were obtained from areas surrounding the ascending aorta and sternotomy incision, respectively. Adipose tissues were collected from patients undergoing myocardial revascularization or mitral valve replacement surgery. Morphological studies with hematoxylin/eosin and immunohistochemical assay were performed in situ to quantify adipokine expression. To analyze adipogenic capacity, adipokine expression, and the levels of thermogenic proteins, adipocyte precursor cells were isolated from periaortic and subcutaneous adipose tissues and induced to differentiation. The precursors of adipocytes from the periaortic tissue accumulated less triglycerides than those from the subcutaneous tissue after differentiation and were smaller than those from subcutaneous adipose tissue. The levels of proteins involved in thermogenesis and energy expenditure increased significantly in periaortic adipose tissue. Additionally, the expression levels of adipokines that affect carbohydrate metabolism, such as FGF21, increased significantly in mature adipocytes induced from periaortic adipose tissue. These results demonstrate that precursors of periaortic adipose tissue in humans may affect cardiovascular events and might serve as a target for preventing vascular diseases.

## 1. Introduction

Several studies have reported that the function of adipose tissue is partly determined by its anatomical location and the influence of adjacent tissues. Perivascular adipose tissue, which surrounds most blood vessels in the body, has recently received much attention [1]. Due to its proximity to the cardiovascular system, perivascular adipose tissue is a determining factor for cardiovascular complications, including atherosclerosis and hypertension [2–4]. Perivascular adipose tissue also secretes adipokines that might affect the function of arteries [5, 6]. Several *in vivo* studies have shown high-calorie diets to reduce adiponectin expression in perivascular adipose tissue, while the levels of proinflammatory cytokines tumor necrosis factor, plasminogen activator inhibitor-1, and monocyte chemoattractant protein-1 increased [3, 4, 7]. Studies in lower mammals have also shown that perivascular fat possesses characteristics of both white adipose tissue (WAT) and brown adipose tissue (BAT) [8, 9]. Periaortic adipose tissue (PAT) is especially important because its anatomical location can affect the characteristics and function of vascular metabolism [10, 11]. Other studies have reported that adipose tissue surrounding the ascending aorta artery expresses proteins involved in energy expenditure, such as uncoupling protein 1 (UCP-1), indicating that it probably is similar to BAT [12], although PAT in the abdominal aorta does not express UCP-1 [13, 14]. Pathophysiological conditions, such as obesity, hypertension, and diabetes, may induce an imbalance in the production of bioactive molecules by perivascular adipose tissue and promote cardiovascular disease [6]. Other recent evidence indicates that cold temperatures can activate PAT and increase thermogenesis, thus improving endothelial function and protecting against atherosclerosis in mice [15].

However, the role of PAT in humans remains elusive. For example, the properties of PAT along the length of different arteries have not yet been defined. Additionally, the presence of metabolically active adipose tissue in the thoracic region remains to be described [16–18]. Although BAT might be present in adults, adipocytes with properties of BAT are characterized as BAT-like or beige [19]. Beige adipocytes might prevent vascular complications in obese patients or those with diabetes mellitus type 2 [20–22]. In this study, we evaluated the morphological, biochemical, and metabolic characteristics of adipose tissue from the ascending aorta (PAT) of patients undergoing myocardial revascularization or mitral valve replacement. The findings were compared with those of subcutaneous adipose tissue (SAT) obtained from the same patients. The increased expression of proteins involved in energy expenditure indicates that the phenotype of PAT resembled beige adipose tissue. Due to the capacity of adipocytes from PAT to increase the thermogenesis activity, this adipose tissue might serve as a target for preventing vascular diseases [9].

## 2. Material and Methods

### 2.1. Patient Characteristics

The study included six women (64 ± 6 years old) and eight men (58 ± 8 years old) with a body mass index (BMI) of 26.6 ± 4.5 kg/m^2^ in women and 27 ± 5.0 kg/m^2^ in men. Only one patient had a diagnosis of type 2 diabetes mellitus. Eight patients were taken to myocardial revascularization, and 3 patients had HTA. Thyroid function, which was assessed by the thyroid-stimulating hormone level, was 3.5 ± 2 mU/L. The average presurgical blood glucose level was 103 mg/dL. Six patients developed postoperative hyperglycemia (41%) with an average of fasting blood glucose of 129 mg/dL. Renal function, which was assessed by the creatinine level, was normal in all patients (Table 1).

All patients were operated on by cardiovascular surgeons at the Cardio-Infantil Foundation in Bogota. PAT was obtained from the area surrounding the ascending aorta, and SAT was removed from the area of the sternotomy incision. All participants reviewed and signed the informed consent prior to the surgical procedure. The study was approved by the ethics committee of the University of La Sabana and Cardio-Infantil Foundation.

### 2.2. Cell Cultures

Adipose tissues were washed in phosphate-buffered saline (PBS) and digested in 250 U/mL type I collagenase, 20 mg/mL bovine serum albumin, and 60 *μ*g/mL gentamicin in PBS for 60 min in a shaking incubator set at 37°C. Thereafter, the cells were centrifuged at 200 ×g for 5 min and incubated in lysis buffer (154 mM NH_4_Cl, 5.7 mM K_2_HPO_4_, and 0.1 mM EDTA (pH 7.3)) for 10 min. The cells were filtered through a 150 *μ*m nylon mesh, followed by centrifugation at 200 ×g for 10 min. To induce proliferation, the cells were cultured in DMEM/F12 supplemented with 15% fetal bovine serum and 50 *μ*g/mL gentamicin for 24 h. After washing, the cells were cultured to confluency in DMEM/F12 supplemented with 10% fetal bovine serum and 50 *μ*g/mL gentamicin.

### 2.3. Induction of Cell Differentiation and Quantification of Lipids

Precursor adipose cells (PAC) were induced to differentiate into mature adipocytes (MAT) in DMEM/F12 supplemented with 66 nM insulin, 1 nM triiodo-L-thyronine, 10 *μ*g/mL transferrin, 0.5 mM isobutyl-methylxanthine, 100 nM dexamethasone, and 1 *μ*M rosiglitazone for 72 h. The medium was then replaced with preadipocyte basal medium containing the same concentrations of insulin, triiodo-L-thyronine, and transferrin, and the cells were cultured for 15 days. The cells were examined under a Carl Zeiss microscope (Germany), and ZEN-lite 2012 software (blue edition) was used to quantify the lipid area. Images were divided into four quadrants, and the surface area of accumulated lipids was quantified in five areas in each quadrant. Data were submitted to analysis, and the differences between the two groups (SAT versus PAT) were evaluated. Adipocyte differentiation was also observed by staining with oil red O, mature adipocyte cells were previously fixed in 10% formaldehyde in PBS for 15 min at 37°C, and the solution of red oil in isopropanol was then added for 2 hours at room temperature. It was subsequently removed and washed with water to remove residual dye. To quantify triglyceride, 0.5 *μ*L mL of isopropanol was added for 5 min, to distain the fat deposits. Absorbance was measured at 510 nm wavelength, and the relative value of triglycerides was determined.

### 2.4. Histological and Immunohistochemical Analysis

SAT and PAT were fixed in 10% formaldehyde in PBS (pH 7.4) at room temperature, dehydrated in ethanol, cleared in xylene, and embedded in paraffin. The tissue blocks were sectioned with a microtome to obtain 4 *μ*m thick sections (American Optical). The sections were incubated with anti-FGF21 (1 : 500, Abcam, Cambridge MA, USA, cat. number ab171941), anti-von Willebrand factor (1 : 100, Novocastra/Leica Biosystems, Wetzlar, Germany), and anti-smooth muscle actin (1 : 100, Novocastra/Leica Biosystems, Wetzlar, Germany) antibodies. The sections were then incubated with anti-rabbit IgG (Novocastra/Leica Biosystems) at 1 : 200 for FGF21 and 1 : 300 for anti-von Willebrand factor for 30 min. The sections were then incubated with 3′,3′-diaminobenzidine hydrochloride (Sigma-Aldrich, St. Louis, MO, USA) for 20 min, followed by three 10 min washes with PBS. Thereafter, the sections were stained with hematoxylin solution (33% solution) for 20 s and washed with PBS for 3 min. The slides were examined under an Olympus BX43 microscope at a magnification of 40x. The results were considered positive when brown precipitates were visible. The capillary number was determined according to the number of adipocytes in the field at a magnification of 40x. Images of adipocytes from SAT and PAT were used to determine the surface area with ImageJ software (National Institutes of Health, Bethesda, MD, USA).

### 2.5. qPCR

Samples of the adipose tissue were frozen in liquid nitrogen, and RNA extraction was performed from 90 mg of SAT and PAT. Samples were treated with lysis solution to isolate RNA using the RNase-Free DNase Kit (Qiagen ref. 79254). SYBER Green was used for real-time reverse transcription polymerase chain reaction (RT-PCR) detection. The purity and the concentration of each sample were measured using a NanoDrop (Thermo Scientific). Briefly, total RNA (1 *μ*g) of each sample was reverse-transcribed in 20 *μ*L using qScript™ cDNA SuperMix (Quanta ref. 84034). Amplification was carried out in a total volume of 10 *μ*L containing 0.5 *μ*M of each primer and 2 *μ*L of 1 : 10 diluted cDNA and PerfeCTa® SYBR® Green FastMix® Low ROX at 1x final concentration (Quanta ref. 84073). qPCR was performed for the detection of PGC-1*α* and UCP1 with primers Fw 5′CTGTGTCACCACCCAAATCCTTAT3′ and Rev 5′TGTGTCGAGAAAAGGACCTTGA3′ and Fw 5′GTGTGCCCAACTGTGCAATG3′ and Rev 5′CCAGGATCCAAGTCGCAAGA3′, respectively. Quantitative analysis was standardized with the corresponding levels of GAPDH primers Fw 5′ACCCACTCCTCCACCTTTGAC3′ and Rev 5′TGTTGCTGTAGCCAAATTCGTT3′ and analyzed by the ΔΔCt method.

### 2.6. Western Blotting

Proteins were isolated in RIPA buffer (Abcam, Cambridge, MA, USA, cat. number ab156034) supplemented with 1 g of protease inhibitors (Roche, cat. number 04693159001). The concentration was measured by the Bradford method, and 50 *μ*g was used for gel electrophoresis. After denaturation at 95°C, the proteins were separated on an 8% polyacrylamide gel and then transferred to PVDF membranes pretreated with absolute methanol for 2 min. The membranes were blocked with 5% skimmed milk in 1x PBS containing 0.1% Tween 20 (PBS-T) and incubated with primary antibodies against proteins involved in thermogenesis or antibodies against adipokines as follows: rabbit anti-PGC-1*α* (1 : 1000, Abcam, Cambridge, MA, USA, cat. number ab54481), rabbit anti-TFAM (1 : 1000, Cell Signaling, Beverly, MA, USA, cat. number ab155117), rabbit anti-CITED1 (1 : 1000, Abcam, Cambridge, MA, USA, cat. number ab87978), rabbit anti-UCP-1 (1 : 1000, Abcam, cat. number ab155117), adiponectin (1 : 3000, Abcam, Cambridge, MA, USA, cat. number ab92501), FGF21 (1 : 2000, Abcam, Cambridge, MA, USA, cat. number ab171941), and FABP4 (1 : 3000, Abcam, Cambridge, MA, USA, cat. number ab92501). The membranes were then incubated with rabbit IgG conjugated to horseradish peroxidase at a dilution of 1 : 5000. The proteins were detected by chemiluminescence using the Luminata Crescendo (Millipore) kit, and images were captured and analyzed with myECL Imager software (Thermo Scientific). Quantitative analysis of three independent experiments was performed by densitometry with the Image Analysis program. Data were analyzed by Student's *t*-test. Differences were considered statistically significant when the value of the mean with standard error was *p* < 0.05.

### 2.7. Immunofluorescent Assays

PAC were cultured on coverslips. The cells were treated with 0.2% Triton X-100 in PBS and fixed in 3.7% formaldehyde in PBS for 15 min. The fixed cells were then permeabilized with 0.5% Triton X-100 in PBS, blocked in 3% bovine serum albumin in PBS for 1 h, and incubated with an anti-PGC-1*α* antibody (1 : 500, Abcam, cat. number ab54481) overnight at 4°C. After washing twice in 0.01% Triton X-100 in PBS, the cells were incubated in Alexa Fluor 488 (1 : 500; Abcam, cat. number 150077) for 1 h. To stain the cell nuclei, the cells were washed twice and mounted in ProLong Diamond Antifade Mountant containing 4′6-diamidino-2-phenylindole (Life Technologies, Eugene, OR, USA). Images were captured with an Eclipse Ni-E microscope (Nikon) and analyzed with ImageJ software.

### 2.8. Statistical Analysis

Data are expressed as mean ± standard deviation (SD). Statistical significance was determined for normally distributed data by using two-tailed Student's *t*-test. Significance was set at ^∗^*p* < 0.05. Statistical analyses were performed with Graph software and SPSS statistics version 22 (IBM).

## 3. Results

### 3.1. Histological Features and the Size of Adipocytes

**Figure 1:**
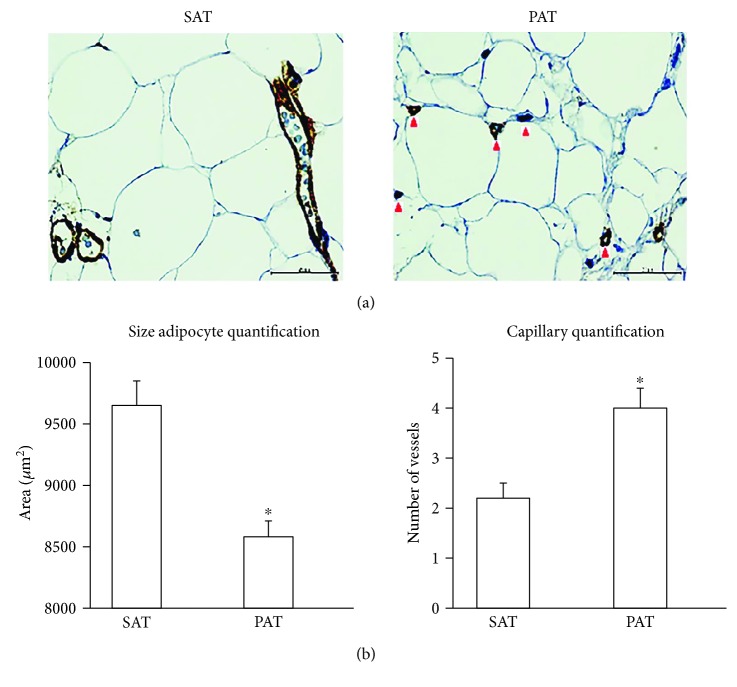
Histological features and the size of adipocytes from PAT and SAT. (a) PAT and SAT were fixed in 10% formaldehyde and used for morphological analysis and the determination of adipocyte size and capillary number. Immunohistochemistry revealed brown intracytoplasmic precipitates only in the intima marked by von Willebrand factor staining (red arrow). (b) Adipocyte size was determined by analyzing five quadrants in each image by ImageJ software. The capillary number was determined by counting 15 random fields in three different plates at a magnification of 40x. ^∗^*p* < 0.05 indicates a statistically significant difference in the size of adipocytes from PAT versus SAT.

**Figure 2:**
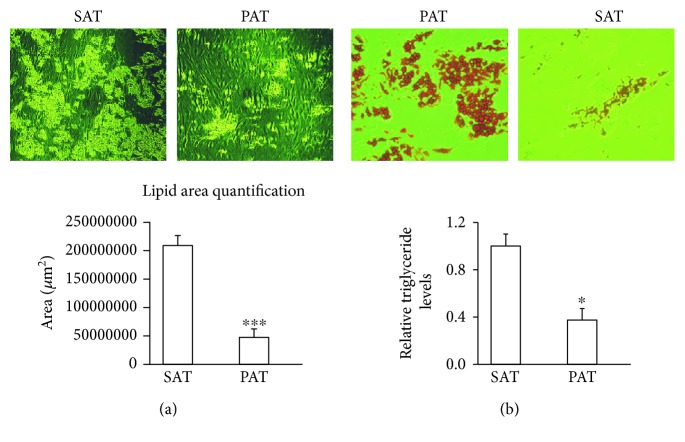
Adipogenic capacity from precursors of PAT and SAT. (a) Precursor cells from PAT and SAT adipocytes were induced to differentiate into mature adipocytes. Images were acquired after 15 days of differentiation. (b) The surface area of accumulated lipids was determined by dividing the image into four quadrants, which were further subdivided into five zones per quadrant. Data are expressed as means ± SD (*n* = 4). ^∗^*p* < 0.05 indicates the differences in the accumulation of triglycerides between SAT and PAT. ^∗∗∗^*p* < 0.001 indicates a statistically significant difference in lipid accumulation in differentiated adipocytes from PAT versus SAT. Relative levels of triglycerides in SAT and PAT were evaluated after the cells reached differentiation. To quantify triglycerides, 1 mL of isopropanol was added for 5 min, to distain the fat deposits. Absorbance was measured at 510 mm wavelength.

SAT and PAT were processed for histological analysis, and the morphology of adipocytes was investigated. Adipocytes from PAT were significantly smaller than adipocytes from SAT (average area: 8608 versus 9592 *μ*m^2^, *p* < 0.05). Compared to adipocytes from SAT, adipocytes from PAT were heterogeneous in size (Figure 1(a)). Several studies have reported increased endocrine activity in highly microvascularized adipose tissue [12, 13]. To determine the capillary number, an anti-von Willebrand Factor antibody was used for immunohistochemistry. The results were considered positive when brown precipitates were visible only in the intima (Figure 1(b)). On the other hand, the capillary number was determined in relation with the number of adipose cells in both tissues at a magnification of 40x (Figures 1(a) and 1(b)). To quantify the surface area of accumulated lipids, adipocyte precursors from SAT and PAT were induced to differentiate for 2 weeks. Consistent with adipocyte size results, the adipogenic capacity was lower in PAT than in SAT; in this determination, a quantification of triglycerides was included (Figures 2(a) and 2(b)).

### 3.2. Expression of Proteins Involved in Thermogenesis in PAT

**Figure 3:**
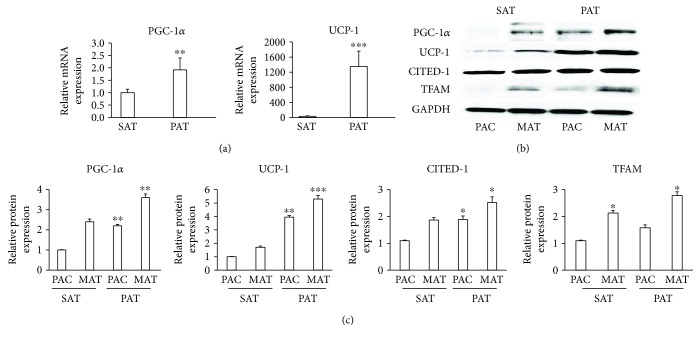
PAT expressed proteins involved in thermogenesis. (a) Samples from SAT and PAT were obtained and immediately frozen in liquid nitrogen. mRNA was extracted and the detection of PGC-1*α* and UCP-1 was performed by qPCR. (b) Precursor adipose cells (PAC) from SAT and PAT were induced to differentiate into mature adipocytes (MAT), and proteins were extracted to quantify the levels of PGC-1*α*, UCP-1, CITED1, and TFAM by Western blotting. (c) Relative intensity of the protein bands was determined by densitometry. Data were normalized to the housekeeping protein GAPDH and expressed as means ± SD (*n* = 4). ^∗^*p* < 0.05, in three different studies, indicates a statistically significant difference in protein levels in precursor adipose cells (SAT versus PAT) and mature adipocyte cells (SAT versus PAT). ^∗∗^*p* < 0.01 indicates the difference between PAT and SAT both in precursor adipocytes and mature adipocytes. ^∗∗∗^*p* < 0.01 indicates the difference between PAT and SAT in mature adipocytes.

Due to striking similarities in the morphology and adipogenic capacity of adipocytes from PAT with the characteristics of brown adipocytes, we isolated and analyzed mRNA and proteins from precursor adipose cells and mature adipocytes from SAT and PAT. Compared to SAT, the levels of PGC-1*α*, UCP-1, CITED1, and TFAM, which play major roles in mitochondrial activity and energy expenditure in brown adipocytes, increased significantly (Figures 3(a), 3(b), and 3(c)).

### 3.3. Expression of PGC-1*α* and the Relationship between SAT and PAT

**Figure 4:**
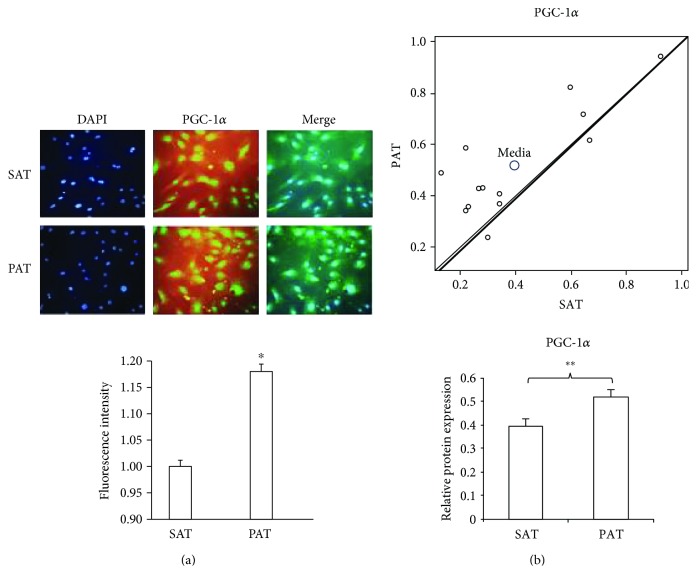
PGC-1*α* expression and the relationship between PAT and SAT. (a) Adipocyte precursor cells were isolated from PAT and SAT, induced to proliferate, fixed in 10% formaldehyde, and stained for the presence of PGC-1*α* by immunofluorescence. The fluorescent intensity was quantified with ImageJ software. Data are expressed as means ± SD. ^∗^*p* < 0.05 indicates the differences in the fluorescent intensity between SAT and PAT. Data correspond to the three different studies. (b) The scatter plot shows the relationship for the PGC-1*α* level in precursor cells from PAT and SAT. Each data point represents a patient. The *y*-axis (*Y*) shows the trend toward PAT expression, and the *x*-axis (*X*) shows the trend toward SAT expression (*n* = 13). The bars indicate intensity levels as determined by densitometry of the bands between SAT and PAT from each patient using paired Student's *t*-test of Western blot analysis of PGC-1*α*. ^∗∗^*p* < 0.01 indicates significative differences in PGC-1*α* protein expression between SAT and PAT.

To confirm the increase in the PGC-1*α* level (Figures 3(a) and 3(b)), we isolated and immunostained precursor cells from PAT and SAT. As expected, the fluorescent intensity was higher in precursor cells from PAT than from SAT, indicating increased expression of PGC-1*α* (Figure 4(a)). To investigate the possible divergence between SAT and PAT, the PGC-1*α* level was quantified by densitometry. The scatter plot shows the differences in the Western blot intensity in each patient showing that the PGC-1*α* level was significantly higher in PAT than in SAT (Figure 4(b)).

### 3.4. Expression of Adipokines in PAT

**Figure 5:**
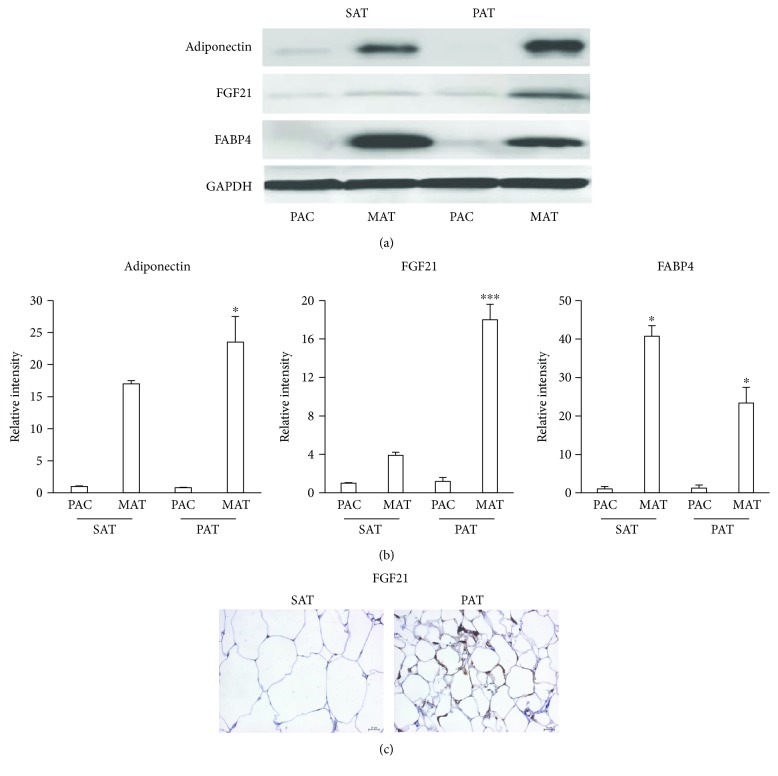
Expression of adipokines in PAT. (a) Adipocyte precursor cells (PAC) from SAT and PAT were induced to differentiate into mature adipocytes (MAT). Proteins were extracted to quantify the levels of adiponectin, FGF21, and FABP4 by Western blotting. (b) Relative intensity of the protein bands was determined by densitometry. Data were normalized to the housekeeping protein GAPDH and expressed as means ± SD (*n* = 4). (c) Specimens were fixed in 10% formaldehyde and stained for the presence of FGF21 by immunohistochemistry. Brown precipitates were indicative of FGF21 within adipocytes. Data were normalized to the housekeeping gene GAPDH. ^∗^*p* < 0.05 indicates differences between PAT mature cells in relation to SAT mature cells. ^∗∗∗^*p* < 0.01 indicates differences between PAT mature cells and SAT mature cells.

One of the biggest challenges involving the anatomical location of adipose tissue is to accurately quantify adipokine levels and to correctly define their metabolic effects [14]. To quantify adipokine levels in different adipose tissues, progenitor cells were isolated and induced to differentiate into mature adipocytes. An increase in the adiponectin level reached significant differences in PAT with respect to SAT. However, the level of FGF21, which regulates glucose metabolism, insulin sensitivity, and energy expenditure [15, 16], increased in adipocytes from PAT compared to those from SAT, reaching the most robust differences (Figures 5(a) and 5(b)). After inducing differentiation, the level of fatty acid-binding protein 4 (FABP4), a marker of lipid accumulation, was significantly higher in adipocytes from SAT than in those from PAT (Figures 5(a) and 5(b)). To confirm the observations about the high expression of FGF21 in adipocytes induced from PAT, we fixed samples from SAT and PAT to perform an immunohistochemistry assay with FGF21 antibody (Figure 5(c)).

## 4. Discussion

In this study, we investigated the structure and function of human perivascular adipose tissue from areas adjacent to the ascending aorta and established that perivascular adipose tissue resembles beige adipose tissue. The levels of proteins involved in energy expenditure (PGC-1*α*, UCP-1, CITED1, and TFAM) increased in adipocytes from PAT compared to those from SAT. Additionally, the levels of adipokines (FGF21 and adiponectin) that have beneficial effects on carbohydrate and lipid metabolism increased in adipocytes from PAT compared to those from SAT.

Adipocytes from PAT were smaller than those from SAT, which is consistent with adipogenic capacity results. After the adipocytes were induced for differentiation, precursor cells from PAT had less lipid accumulation than those from SAT (Figure 2). Capillary vascularization is higher in PAT than in SAT, indicating that it has increased metabolic activity. Previous studies have described the importance of the microvasculature in the control of metabolic function. For example, studies in obese mice have reported a reduction in capillary density accompanied by hypoxia in WAT, contrary to the highly vascularized and thermogenic BAT [5, 23, 24]. Although the number of patients was limited and the differences were not significant, a reduction of TFAM was observed in obese patients. The increase in postoperative glycaemia was not related to morphological or functional change in the periaortic fat, nor was the observed differences in relation to sex.

In recent years, researchers have aimed to characterize the role of the perivascular adipose tissue in cardiovascular metabolism. Its anatomical location may affect its phenotype. For example, epicardial and pericoronary adipocytes are brown-like [17–19]. Studies in rats have reported that adipose tissue from an area adjacent to the ascending aorta possesses morphological and functional characteristics that are identical to those of BAT, while adipose tissue from an area surrounding the abdominal aorta is similar to WAT [25]. Additionally, the increased levels of PGC-1*α*, UCP-1, CITED1, and TFAM in adipocytes isolated from adipose tissue surrounding the ascending aorta stimulate mitochondrial activity and heat production. The expression of these proteins is similar to what is observed in previous studies into adipocyte beige; in fact, CITED 1 has been shown as a specific marker of beige adipocytes [26, 27]. It is possible that the mechanism of the development of perivascular adipose cells is like arterial smooth muscle cells. An *in vivo* study has shown that ectopic expression of the transcriptional coregulator PR domain containing 16 (PRDM16) induces the development of smooth muscle cells into beige adipose cells, and the PPAR*γ* deletion ablates the perivascular depot [28]. It is also possible that brain and atrial natriuretic peptides, which induce differentiation of white adipose cells to brown adipose cells, affect perivascular adipocytes at the level of the ascending aorta [29].

Adipocytes with BAT-like properties showed a better capacity for glucose utilization and circulating lipid management, which might be mediated by increased expression of FGF21 and adiponectin, because both proteins can regulate the function of the aorta. Adiponectin reduces muscle cell proliferation *in vitro* and *in vivo* via an AMPK-dependent pathway and induces vasodilatation by promoting eNOS activity [30, 31]. The production of FGF21 by BAT also increases the transdifferentiation of WAT into brown-like adipocytes. FGF21 might also reduce the levels of triglycerides and LDL-C cholesterol, induce adiponectin production, and increase insulin activity [32, 33]. These effects on brown-like adipocytes should be further investigated to determine if different circumstances in humans, such as obesity, lipid alterations, and diabetes mellitus type 2, can affect thermogenesis and metabolism in these cells [19, 34]. Adipocytes from BAT can protect the cardiovascular system from atherosclerosis by preventing lipid accumulation and vascular inflammation. Mice subjected to a diet high in cholesterol under thermoneutral conditions develop atherosclerosis, while mice exposed to a cold environment activate thermogenesis and generate a protective effect against vascular damage. In this study, we found that PAT expresses proteins, such as the adipokine FGF21, which activates thermogenesis and confers a brown-like phenotype. Our data has provided insights into the metabolism of adipose tissue at the level of the ascending aorta in humans because PAT possesses a BAT-like phenotype. Additional studies are needed to determine whether modulation of this phenotype can prevent pathological events such as atherosclerosis.

## Figures and Tables

**Table 1:** Blood biochemical levels of the patients.

Patient	Sex	Age	BMI	HbA1c	Glycem pre-Qx.	Glycem post-Qx.	T. cholest.	HDL cholest.	TSH	Creat.
1	Female	66	22	5.50	92	99	159	45	4.69	0.8
2	Female	76	29	7.70	123	140	121	38	3.30	1.3
3	Female	79	27	5.20	96	111	205	41	3.90	1.3
4	Female	71	32	5.60	100	105	253	49	2.03	0.5
5	Female	54	22	5.30	92	110	220	29	4.20	0.8
6	Female	48	20	5.60	97	145	210	41	3.80	1
7	Male	59	31	5.60	99	98	190	28	7.37	1.5
8	Male	57	30	5.30	88	150	177	30	2.03	0.9
9	Male	39	21	5.30	92	94	218	42	1.90	0.7
10	Male	57	28	5.92	103	145	189	29	5.88	1.2
11	Male	65	27	5.40	97	130	210	33	1.31	0.7
12	Male	74	22	5.50	95	170	193	42	2.59	0.7
13	Male	52	29	5.30	84	81	193	32	3.00	0.9
14	Male	58	27	5.80	83	86	146	40	3.36	1.2

BMI: body mass index; HbA1c: glycosylated haemoglobin; Glycem pre-Qx.: glycaemia before surgery; Glycem post-Qx.: glycaemia after surgery; T. cholest.: total cholesterol; HDL cholest.: high-density cholesterol; TSH: thyrotropin; Creat.: serum creatinine.
